# The immunosuppressive factors IL-10, TGF-β, and VEGF do not affect the antigen-presenting function of CD40-activated B cells

**DOI:** 10.1186/1756-9966-31-47

**Published:** 2012-05-16

**Authors:** Alexander Shimabukuro-Vornhagen, Andreas Draube, Tanja M Liebig, Achim Rothe, Matthias Kochanek, Michael S von Bergwelt-Baildon

**Affiliations:** 1 Cologne Interventional Immunology (CII), University Hospital of Cologne, Cologne, Germany; 2 Critical Care Unit, Department I of Internal Medicine, University Hospital of Cologne, Cologne, Germany; 3 Cologne Interventional Immunology (CII), Department I of Internal Medicine, University Hospital of Cologne, Kerpener Str. 62, 50924, Cologne, Germany

**Keywords:** CD40-activated B cells, Antigen-presenting cells, IL10, TGF-β, VEGF, Tumor immunotherapy

## Abstract

**Background:**

Progress in recent years strengthened the concept of cellular tumor vaccinations. However, a crucial barrier to successful cancer immunotherapy is tumor-mediated immunosuppression. Tumor-derived soluble factors such as IL-10, TGF-β, and VEGF suppress effector cells either directly or indirectly by disruption of dendritic cell (DC) differentiation, migration and antigen presentation. Human B cells acquire potent immunostimulatory properties when activated via CD40 and have been shown to be an alternative source of antigen-presenting cells (APCs) for cellular cancer vaccines. Nevertheless, in contrast to DCs little knowledge exists about their susceptibility to tumor derived immunosuppressive factors. Thus, we assessed whether IL-10, TGF-β, or VEGF do affect key aspects of the immunostimulatory function of human CD40-activated B cells.

**Methods:**

Cell surface expression of adhesion and costimulatory molecules and the proliferation capacity of CD40-activated B cells were compared to untreated controls by flow cytometry. Migration towards important chemokines of secondary lymph organs was measured with or without exposure to the immunosuppressive cytokines. Finally, an influence on T cell stimulation was investigated by allogeneic mixed lymphocyte reactions. For statistical analysis Student’s *t* test or two-way analysis of variance followed by Bonferroni's post-hoc test was used to compare groups. *P* values of <0.05 were considered statistically significant.

**Results:**

Neither cell adhesion nor the expression of MHC class II and costimulatory molecules CD80 and CD86 was inhibited by addition of IL-10, TGF-β, or VEGF. Likewise, the proliferation of CD40-activated B cells was not impaired. Despite being exposed to IL-10, TGF-β, or VEGF the B cells migrated equally well as untreated controls to the chemokines SLC and SDF-1α. Most importantly, the capacity of CD40-activated B cells to stimulate CD4^+^ and CD8^+^ T cells remained unaffected.

**Conclusion:**

Our findings suggest that key immunostimulatory functions of CD40-activated B cells are resistant to inhibition by the immunosuppressive factors IL-10, TGF-β, and VEGF. This supports considerations to use ex vivo generated CD40-activated B cells as a promising alternative or additional APC for cellular immunotherapy, especially in settings where these immunosuppressive cytokines are present in tumor environment.

## Background

The immune system plays an important role in the control of tumor development and progression. Thus, since decades immunotherapeutic strategies aim to exploit the ability of the immune system to detect and destroy tumor cells. One of the most promising concepts is the use of antigen-presenting cells (APCs) as cellular adjuvants for tumor vaccination. Especially, dendritic cells (DCs) have been identified as the ideal APC source due to their natural antigen-processing and presenting functions, their migratority capacities and the ability to activate naïve T cells [1]. However, a general barrier to successful cancer immunotherapy is the tumor-induced immunosuppression which is mainly mediated by tumor-derived soluble factors in the tumor microenvironment [2,3]. This is also true for APC-based tumor vaccinations strategies [4].

Among the most well-known and best characterized tumor-derived immunosuppressive molecules are interleukin-10 (IL-10) [5,6], transforming growth factor-beta (TGF-β) [7,8], and vascular endothelial growth factor (VEGF) [9,10]. An important mechanism by which IL-10, TGF-β, and VEGF counteract the development of an anti-tumor immune response is through inhibition of DC differentiation, maturation, trafficking, and antigen presentation [6,11].

In recent years the antigen-presenting function of B lymphocytes has gained increasing attention. Accumulating evidence demonstrates that B cells serve many functions in the immune response beside antibody mediated mechanisms [12]. Cytokine production and antigen-presentation are important mechanisms by which B lymphocytes contribute to both to immunity and immune pathology [13-16]. Activated antigen-presenting B cells have been shown to efficiently induce both CD4^+^ and CD8^+^ T cells responses in vitro and in vivo [17-20]. Therefore, many research groups are currently evaluating B cell-based vaccines as an alternative to DC-based vaccines for cancer immunotherapy [18,19,21-27].

CD40-activated B cells can be prepared at relatively low costs as a highly pure homogenous population that can be expanded from small amount of peripheral blood even from cancer patients [28]. However, it is not known whether tumor-derived immunosuppressive factors affect the antigen-presenting capacity of CD40-activated B cells in a similar fashion as in DC. We therefore studied the effect of IL-10, TGF-β, and VEGF on the phenotype, migratory ability, and T cell stimulatory capacity of CD40-activated B cells in vitro.

## Methods

### Flow cytometry

Immunophenotypic analysis was performed using fluorescence-activated cell sorting (FACS) according to standard protocols. The cells were analyzed on a FACSCanto flow cytometer (BD Biosciences, Heidelberg, Germany). Antibodies against CD19, CD80, CD86, HLA-DR, CD3, and CD25 were purchased from BD Pharmingen (Heidelberg, Germany).

### Generation of CD40-activated B cells and cell culture

CD40-B cells were generated as described previously [29]. In brief, whole PBMC were cultured on irradiated NIH3T3 fibroblasts transfected with human CD40 ligand (tCD40L) in the presence of recombinant human interleukin-4 (2 ng/ml; R&D Systems, Minneapolis, MN, USA) and clinical-grade cyclosporin A (CsA, 5·5 × 10^−7^ M; Novartis, Basel, Switzerland) in Iscove's modified Dulbecco's medium (IMEM; Invitrogen, Karlsruhe, Germany) supplemented with 10% pooled human serum. The expanding cells were transferred onto freshly prepared tCD40L cells and fed with cytokine-replenished medium without CsA every 3–4 days. After 2–3 weeks in culture the CD40-activated B cells had a purity of >95 % and were used for the experiments. Therefore they were cultured for 4 days in the presence of 40 ng/ml IL-10, 10 ng/ml TGF-β, 20 ng/ml VEGF or vehicle as a control. For these concentrations the inhibitory effects on APC functions of DCs have been demonstrated previously [11]. Prior to use the activity of IL-10, TGF-β, and VEGF at the given concentrations was tested by assessing their inhibitory effect on DC maturation and for IL-10 and TGF-β additionally on T cell proliferation.

### In vitro migration assay

To assess B cell migration, 5 × 10^5^ CD40-B cells were transferred into the upper chamber of 5-μm pore size transwell plates (Costar, Cambridge, MA, USA). Varying amounts of the chemokines SDF-1α and SLC (R&D Systems) were added to the lower chamber. After 2 hours at 37°C, the number of cells that had migrated into the lower chamber was determined using a hemacytometer.

### T cell proliferation assay

Untouched CD4^+^ T cells and CD8^+^ T cells were obtained from buffy coats by negative selection using Rosette Sep® (StemCell Technologies) human CD4^+^ and CD8^+^ T cell enrichment cocktails according manufacturers’ instructions. Prior to allogeneic mixed lymphocyte reactions (MLR) the CD4^+^ and CD8^+^ T cells were labeled with carboxyfluorescein diacetate succinimidyl ester (CFSE, Molecular Probes) according to standard protocols. A total of 1 x10^5^ CFSE-labeled CD4^+^ or CD8^+^ T cells were co-incubated with allogeneic CD40-B cells as stimulators at different B to T cell ratios ranging from 1:1 to 1:20. After 5–7 days proliferation was assessed by flow cytometry.

### Statistical analysis

Data are reported as means ± standard deviation unless stated otherwise. Student’s *t* test or, where appropriate, two-way analysis of variance followed by Bonferroni's post-hoc test was used to compare groups. *P* values of <0.05 were considered statistically significant.

## Results

### Phenotype of CD40-activated B cells

**Figure 1:**
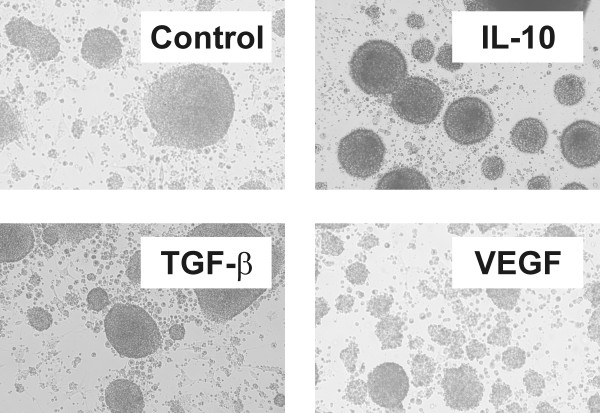
**Morphology of CD40-activated B cells.** Cluster formation of CD40-activated B cells through homotypic adhesion is not affected by IL-10, TGF-β, or VEGF for 4 days.

Upon activation via CD40 B cells upregulate the expression of MHC class II, costimulatory molecules, and adhesion molecules and as a consequence they acquire potent T-cell stimulatory activity. We therefore first studied the effect of IL-10, TGF-β, and VEGF on the morphology and cell surface expression of HLA-DR and costimulatory molecules of CD40-activated B cells. The upregulation of adhesion molecules such as ICAM-1 results in the formation of round clusters through homotypic adhesion of activated B cells. As shown in Figure 1 IL-10, TGF-β, and VEGF had no impact on cluster formation of CD40-activated B cells.

**Figure 2:**
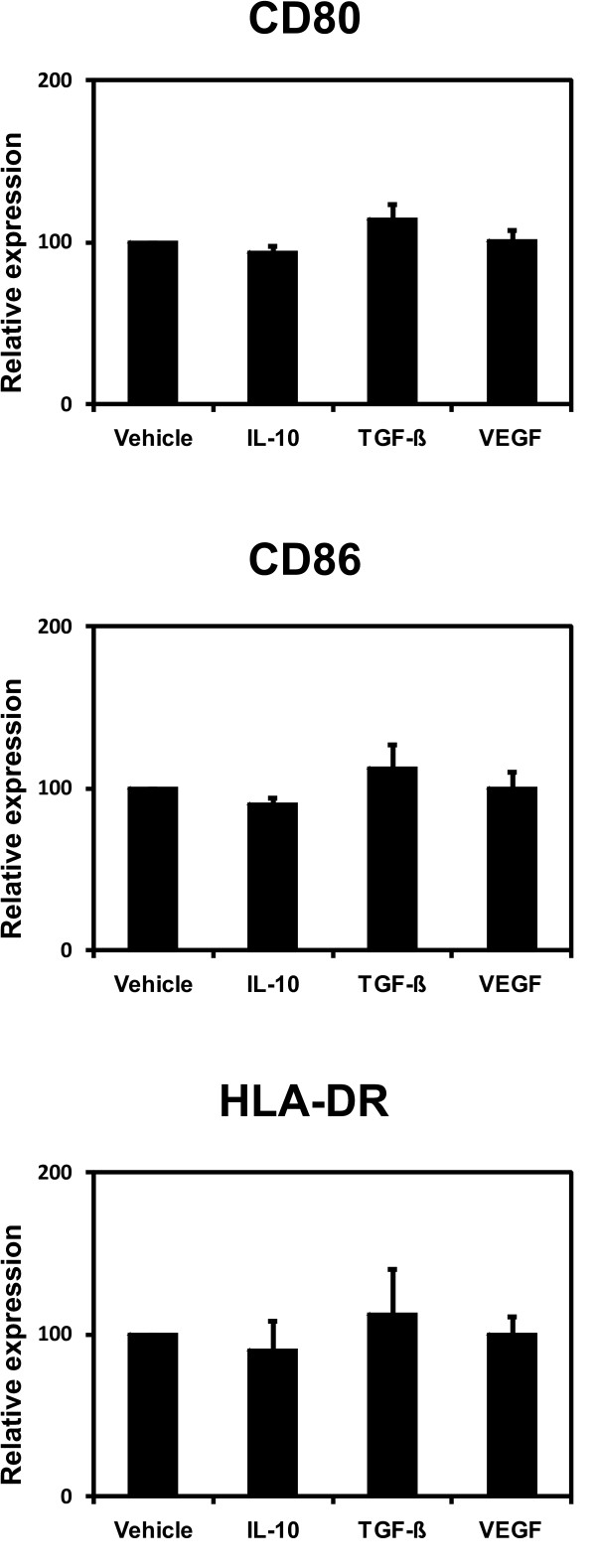
**Phenotype of CD40-activated B cells.** CD40-activated B cells were cultured on CD40L-expressing NIH3T3 fibroblasts in the presence of 40 ng/ml IL-10, 10 ng/ml TGF-β, 20 ng/ml VEGF or vehicle. After 4 days in culture the surface expression of HLA-DR and the costimulatory molecules CD80 and CD86 by CD40-activated B cells was assessed by flowcytometry. Shown is the mean fluorescence intensity relative to vehicle-treated CD40-activated B cells. The bar graph shows the means of 6 independent experiments ± SD.

For the same activation protocol used in this work we have repeatedly shown a strong upregulation of CD80, CD86 and HLA-DR both for B cells of healthy donors and of cancer patients [28,29]. Thus, we used the expression levels of vehicle treated CD40-activated B-cells as baselines and these were compared to the expression levels of cells exposed to the immunosuppressive cytokines. In a series of experiments no statistically significant differences between CD40-activated B cells treated with IL-10, TGF-β, or VEGF in comparison to controls were observed (Figure 2).

### Proliferation of CD40-activated B cells

Activation via CD40 induces proliferation of B cells. We assessed whether the proliferation was inhibited by any of the three immunosuppressive factors. Table 1 summarizes the results of the proliferation of CD40-activated B cells cultured in the presence of either IL-10, TGF-β, or VEGF. After four days the cells were removed from the wells and the proliferation was determined by counting. TGF-β and VEGF exerted no effect on the proliferation of B cells activated through CD40. Consistent with previous reports we found that IL-10 enhanced the expansion of CD40-activated B cells [30].

**Table 1:** Proliferation of CD40-activated B cells

	**Mean (%)**	**SD**	**p**
**Control**	**197**	**+/− 52**	**-**
**IL-10**	**301**	**+/− 106**	**< 0.01**
**TGF-β**	**222**	**+/− 95**	**Not significant**
**VEGF**	**197**	**+/− 70**	**Not significant**

Means of the relative increase in cell number of 8 experiments.

### Migratory ability

**Figure 3:**
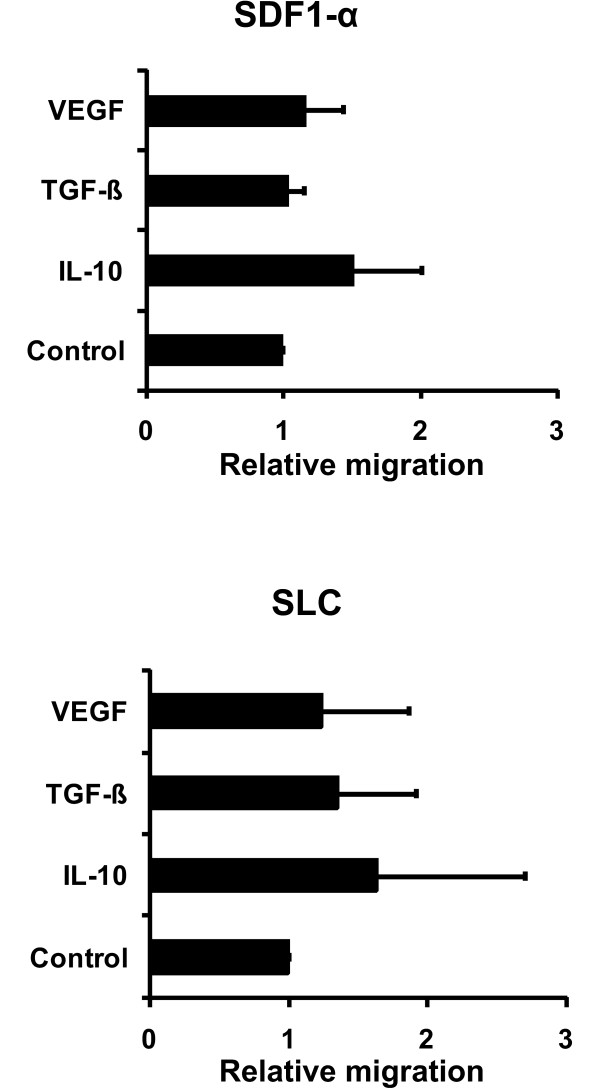
**Migratory ability of CD40-activated B cells.** 5 × 10^5^ CD40-B cells were added to the upper chamber transwell plates. Varying amounts of the chemokines SDF-1α and SLC (R&D Systems) were added to the lower chamber. After 2 hours the cells that had migrated into the lower chamber were counted with a hemacytometer. The migration index is calculated relative to vehicle-treated controls. Shown are the means of 4 independent experiments ± SD.

Migration of APCs to the secondary lymphoid organs is essential for the induction of CD4^+^ and CD8^+^ T cell responses. For CD40-activated B cells of healthy donors and of cancer patients the migration capacity has been shown [28,31]. We thus studied the influence of IL-10, TGF-β, and VEGF on the migratory ability of CD40-activated B cells towards the important lymph node homing cytokines SDF-1α and SLC in vitro. We used the migration of vehicle treated CD40-activated B cells as controls (relative migration =1). The T cell migration of CD40-activated B cells treated with IL-10, TGF-β, or VEGF in comparison to these controls are shown in Figure 3. CD40-activated B cells migrated equally well towards SDF-1α and SLC independent of whether they were treated with vehicle, IL-10, TGF-β, or VEGF.

### T cell stimulation by CD40-activated B cells

**Figure 4:**
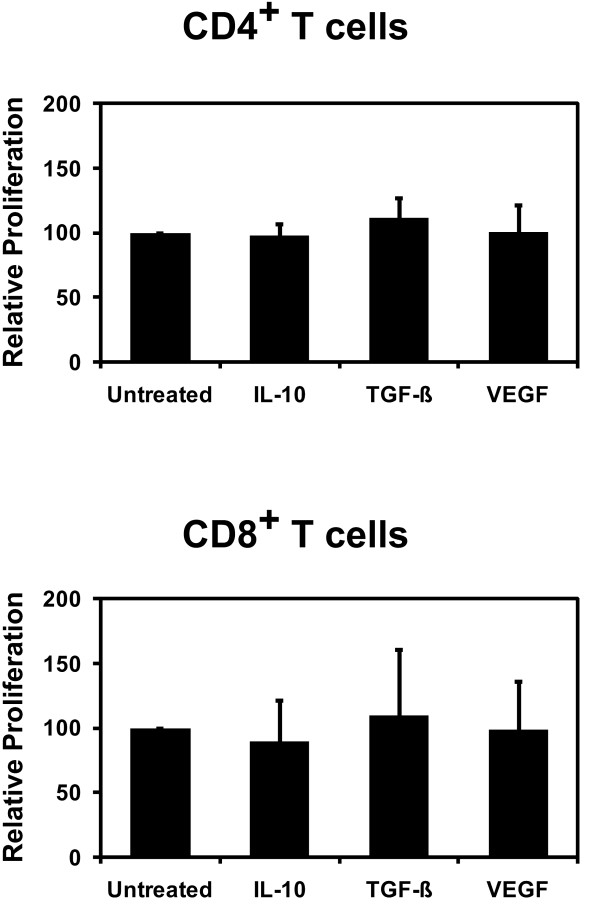
**T cell-stimulatory capacity of CD40-activated B cells.** 1 x 10^4^ treated and control CD40-activated B cells were incubated with 1 x 10^5^ CFSE-labeled allogeneic T cells. After 5 days the proliferation of the allogeneic CD4^+^ and CD8^+^ T cells was assessed by flow cytometery. IL-10, TGF-β, or VEGF did not inhibit the proliferation of allogeneic CFSE-labeled CD4^+^ (n = 8) and CD8^+^ T cells (n = 5) in response to CD40-activated B cells. The data shown represents the means of the relative percentage of CD25^+^ proliferating, i.e. CFSE^low^, T cells ± SD.

In order to assess the impact of tumor-derived immunosuppressive factors on the T cell-stimulatory capacity of CD40-activated B cells we compared the ability of CD40-activated B cells which were treated with IL-10, TGF-β, or VEGF to induce the proliferation of CFSE-labeled CD4^+^ or CD8^+^ T lymphocytes from healthy HLA-mismatched donors. Figure 4 shows the result of the CFSE-proliferation assays comparing vehicle controls with CD40-activated B cells which were exposed to IL-10, TGF-β, or VEGF. We did not observe statistically significant differences in the proliferation of CD4^+^ or CD8^+^ T cells between the controls and CD40-activated B cells which were cultured in the presence of 40 ng/ml IL-10, 10 ng/ml TGF-β, or 20 ng/ml VEGF. Therefore, neither IL-10, TGF-β, nor VEGF was able to inhibit the capacity CD40-activated B cell to activate CD4^+^ or CD8^+^ T lymphocytes.

## Discussion

Due to a growing body of knowledge about immunosurveillance – and loss thereof – anti-tumor immunotherapy has been refined [32]. Nevertheless, especially results of APC-based tumor vaccination trials often have often not met the high expectations. Lack of efficacy mainly originates from well-defined tumor escape mechanisms [2,3,33]. Tolerizing conditions of the tumor environment are mainly driven by tumor or bystander cell derived cytokines inducing tolerogenic DC, e.g. by triggering myeloid DC B7-H1 expression [34], and by recruitment of regulatory T cells [35], myeloid-derived suppressor cells (MDSCs) and mesenchymal stroma cells (MSCs) [36]. IL-10, TGF-β, and VEGF all have been identified as key factors that mediate the inhibitory action of the tumor microenvironment. Their serum levels are frequently increased in cancer patients and the tumor tissues of many cancer types are enriched for these immunosuppressive factors [37-39]. The main activity of IL-10 is related to downregulation of T cell function, which occurs predominantly through indirect mechanisms involving APCs [40]. IL-10 has been shown to impair antigen-presentation by DCs through reduction of the cell surface expression of adhesion and costimulatory molecules as well as MHC class II. Furthermore, IL-10 promotes DC apoptosis and inhibits DC migration to the secondary lymphoid organs [41,42]. DCs isolated from transgenic mice that over-express IL-10 have a defect in antigen presentation and decreased capacity to induce T cell activation. Conversely, in IL-10-deficient tumor-bearing mice the defect in DC function was reversed [43]. As a consequence IL-10-conditioned DCs are tolerogenic and induce T cell anergy [6,44]. Like IL-10 TGF-β prevents the trafficking of DCs to the lymph nodes [45]. In addition, TGF-β impairs the maturation of DCs and thereby leads to the accumulation of immature DCs with the ability to generate regulatory T cells [8,46]. VEGF also inhibits DC maturation leading to an accumulation of immature DCs with impaired APC function within the tumor microenvironment and the tumor-draining lymph nodes [9]. Consequently, inhibition of TGF-β, IL-10, or VEGF signaling improves DC function and enhances the efficacy of tumor vaccines [47-49]. Another strategy to address these tumor escape mechanisms in cellular tumor vaccinations is the use of alternative APC sources. In this context human CD40-activated B cells have gained increasing interest.

We and others have previously shown that CD40-activated B cells are equipped with a profile of chemokine receptors that are required for the homing to the secondary lymphoid organs [31]. Furthermore, CD40-activated B cells are potent antigen-presenting cells and are able to prime both CD4^+^ and CD8^+^ T cells in vitro. The capacity of CD40-activated B cell-based cancer vaccine to induce CD4^+^ and CD8^+^ T cell responses also has been shown in vivo in mice and a large animal model in dogs [22,25,27,50,51]. An important advantage of CD40-activated B cells is that they can be highly expanded at relatively low cost from small amounts of peripheral blood even from cancer patients [21,28]. Nevertheless, it has also has been proposed that their APC functions have to be further evaluated in more detail before they are used in therapeutic vaccinations [52].

It is known that IL-10, TGF-β, and VEGF play important roles in the regulation of B cells. TGF-β specifically induces the class switch to IgA while IL-10 promotes switching to IgA, IgG, and IgE [53]. TGF-β furthermore induces apoptosis in resting B cells and inhibits B cell proliferation [54]. VEGF leads to the accumulation of B cells in the spleen [55]. However, compared to DCs the influence of these immunosuppressive cytokines on CD40-activated B cells is poorly characterized. We therefore studied the effects of IL-10, TGF-β, and VEGF on crucial steps in the generation of a T cell-mediated immune response in vitro. Neither TGF-β nor VEGF had a significant effect on B cell proliferation. Exposure to IL-10 on the other hand increased the expansion of B lymphocytes. The migratory ability of B cells remained unchanged after exposure to all the three immunosuppressive factors. Even though it was previously reported that IL-10 impairs the motility of murine and human B cells [56] the activation by CD40 seems to protect B cells from the inhibitory effect of IL-10. For TGF-β our findings supports assumptions from previous reports that some of the immunosuppressive effects on B cells can be blocked by CD40 signaling [57,58]. Thus, with the notable exception of the enhancing effect of IL-10 on B cell proliferation important APC functions of CD40-activated B cells are not affected by IL-10, TGF-β, or VEGF.

## Conclusion

In summary, our results show that at least in vitro the APC function of CD40-activated B cells is highly resistant to inhibition by the immunosuppressive factors IL-10, TGF-β, and VEGF, which have been shown to play an important role in the immunosuppressive microenvironment of many tumors and to interfere with the differentiation and APC function of DCs. Thus, ex vivo generated CD40-activated B cells are well suited as APCs for cellular vaccines. They represent a promising alternative or additional APC for cellular immunotherapy, especially in settings where the above cytokines are present in the tumor microenvironment.

## Misc

Alexander Shimabukuro-Vornhagen and Andreas Draube authors contributed equally to this work.

## Competing interests

The authors declare that they have no competing interests.

## Authors’ contributions

ASV and AD made substantial contributions to conception and design as well as to the interpretation of the data and drafted the manuscript. TML and ASV carried out the experiments. TML, AR and MK contributed to conception, the interpretation of the data and assisted to draft the manuscript. MBB conceived of the study, participated in its design and coordination and helped to draft the manuscript. All authors read and approved the final manuscript.
